# A deep learning-based framework for lung cancer survival analysis with biomarker interpretation

**DOI:** 10.1186/s12859-020-3431-z

**Published:** 2020-03-18

**Authors:** Lei Cui, Hansheng Li, Wenli Hui, Sitong Chen, Lin Yang, Yuxin Kang, Qirong Bo, Jun Feng

**Affiliations:** 1 Department of Information Science and Technology, Northwest University, Xi’an, China; 2 The College of Life Sciences, Northwest University, Xi’an, China

**Keywords:** Cell detection, Deep learning, Feature learning, Survival analysis

## Abstract

**Background:**

Lung cancer is the leading cause of cancer-related deaths in both men and women in the United States, and it has a much lower five-year survival rate than many other cancers. Accurate survival analysis is urgently needed for better disease diagnosis and treatment management.

**Results:**

In this work, we propose a survival analysis system that takes advantage of recently emerging deep learning techniques. The proposed system consists of three major components. 1) The first component is an end-to-end cellular feature learning module using a deep neural network with global average pooling. The learned cellular representations encode high-level biologically relevant information without requiring individual cell segmentation, which is aggregated into patient-level feature vectors by using a locality-constrained linear coding (LLC)-based bag of words (BoW) encoding algorithm. 2) The second component is a Cox proportional hazards model with an elastic net penalty for robust feature selection and survival analysis. 3) The third commponent is a biomarker interpretation module that can help localize the image regions that contribute to the survival model’s decision. Extensive experiments show that the proposed survival model has excellent predictive power for a public (i.e., The Cancer Genome Atlas) lung cancer dataset in terms of two commonly used metrics: log-rank test (*p*-value) of the Kaplan-Meier estimate and concordance index (*c*-index).

**Conclusions:**

In this work, we have proposed a segmentation-free survival analysis system that takes advantage of the recently emerging deep learning framework and well-studied survival analysis methods such as the Cox proportional hazards model. In addition, we provide an approach to visualize the discovered biomarkers, which can serve as concrete evidence supporting the survival model’s decision.

## Background

Lung cancer is the leading cause of cancer-related deaths in both men and women in the United States. An estimated 158,080 Americans died from lung cancer in 2016, accounting for approximately 27% of all cancer deaths^1^. The five-year survival rate of lung cancer is 17.7*%*, which is lower than that of many other leading cancers, such as colon cancer (64.4*%*) and breast cancer (89.7*%*). There are two main types of lung cancer: small cell lung cancer (SCLC) and non-small cell lung cancer (NSCLC). NSCLC accounts for the majority of lung cancer (80*%*−85*%*) and has two major subtypes: adenocarcinoma (AC), representing approximately 40%, and squamous cell carcinoma (SC), representing approximately 25*%*−30*%*^2^. Accurate survival analysis is essential for personalized treatment management and prognosis. For example, closer follow-up and more aggressive treatment might benefit patients with poorer prognoses [1].

### Cell localization

Currently, histopathology images serve as the golden standard for lung cancer diagnosis and are primarily evaluated by pathologists or doctors. However, this process is labor intensive, time consuming, and subject to high inter-observer variability. Recently, an automated histopathological analysis system [2] has been shown to be able to provide accurate, consistent, and valuable decision support for the diagnosis of different diseases, such as breast cancer [3], pancreatic neuroendocrine tumors [4], lymphoma [5], and lung cancer [1, 6–8]. With the emergence of deep learning methods that have achieved great successes in computer vision [9–12], in this work, we aim to develop a deep learning-based lung cancer survival analysis system that can provide accurate prediction of patient survival outcomes and identify important image biomarkers.

Pathologists make diagnostic decisions based on cellular and inter-cellular level morphology, and thus accurate cell localization is a prerequisite step for lung cancer survival analysis. Since cells usually exhibit circular or approximately circular shapes and their sizes fall within a relatively small range, many methods [13–15] are designed to fully utilize this prior information. These methods primarily consist of three steps: cell confidence map generation, cell center localization, and optional post-processing. Here, we refer to a cell confidence map as an intermediate image transform which highlights cell centers. For example, in [14], Byun et al. first apply a Laplacian of Gaussian (LoG) filter with a fixed scale for locating nuclei on retinal images, assuming that the cell size is known a priori and cell detection can be achieved by locating the maximum filter response in a neighborhood with a predefined size. Observing that many cells exhibit round shapes, Veta et al. [16] used a fast radial symmetry transform (FRST) [17] to generate cell confidence maps. Following this idea of radial symmetry-based voting, Parvin et al. [18] have proposed an iterative voting approach based on (weakly) radial symmetries, which is adaptive to geometric perturbation and can handle elliptical objects. However, the iterative procedure in [18] is computationally expensive. To address this issue, Qi et al. [15] have proposed single-pass voting for cell detection which performs only one round of voting and computes the final cell centers by applying mean shift clustering [19] to the vote image.

Recently, deep learning-based models, especially convolutional neural networks (CNNs), have attracted particular interest [9–12] and achieved state-of-the-art performance in various vision tasks, such as image classification [20, 21], object detection [22, 23], segmentation [24] and so on. Great successes of applying CNN to medical image analysis have also been reported [25–28]. In [25], Ciregan et al. applied a deep CNN for automatic mitotic cell detection in breast cancer histology images. In [27], Song et al. first computed a pixel-wise coarse segmentation with CNN and achieved the final nuclei locations using a fast min-cut/max-flow graph inference algorithm [29]. In both [25] and [27], the CNN models are applied to testing images in a sliding-window manner for pixel-wise classification, which is computationally expensive. Recently, Long et al. [30] proposed a fully convolutional network (FCN). In contrast with conventional CNN methods [9–12], an FCN is trained in an end-to-end manner and can produce output maps with the same size as the inputs, and thus it is both asymptotically and absolutely efficient [30].

### Survival analysis

Survival analysis is a well-studied field in health statistics research which aims at predicting the time until the occurrence of an event of interest, such as onset of a disease, tumor recurrence, death after some treatment intervention, etc. The time between the beginning of follow-up and the occurrence of the event is called *survival time* or *failure time*. In survival analysis, one important issue to be considered is the censoring problem. For example, this occurs when a subject is not followed up during the study period or does not experience the event of interest before the study ends. Since the exact survival times of the censored subjects are unknown, and they account for a large portion of the data, standard statistical methods such as linear regression are not suitable for survival time data. In survival analysis, the most commonly used method is the Cox proportional hazards model [31]. Other methods include the Kaplan-Meier estimate [32] for calculating the survival probability and the log-rank test [33] for comparing the survival outcomes of two or more subject groups.

Recently, there have been several works published regarding survival analysis of lung cancer using pathological image features [1, 6–8]. In [6], Wang et al. have proposed three groups of image morphological features (geometry features, pixel intensity statistics, and texture features) extracted from the segmented cell regions, and the Cox proportional hazards model is used to select image features that are correlated with patient survival outcomes. Similarly, Yao et al. [7] have enhanced the three groups of image features by including the spatial distributions of cell subtypes (tumor, lymphocyte, stromal) and have built a separate survival model for each of the two major subtypes of NSCLC: adenocarcinoma and squamous cell carcinoma. In [1], the cells are first segmented out using the Otsu threshold selection method [34], and then a total of 9,879 quantitative features are extracted from each image patch using CellProfiler [35]. They all achieved success in building a powerful survival model and finding valuable biomarkers. For example, in [6], pixel intensity and texture features are found to be correlated with survival outcomes; in [7], image features related with cell subtype distributions, cell shape and granularity are selected; in [1], Zernike shape, texture and radial distribution of pixel intensity are among the top prediction features.

However, the aforementioned methods [1, 6, 7] face several limitations. First, they all require cell segmentation as a prerequisite step. As opposed to cell detection, accurate, robust and efficient cell segmentation remains a challenging task because 1) there exist significant variations in intra- and inter-cellular intensity, especially for cancer cells across different patients, and 2) cells are often clustered into clumps, such that they might partially overlap with one another. Inaccurate cell segmentation might harm the discriminative power of some morphological features, such as cell granularity or shape. Second, the cell features are currently handcrafted: thus, they are error-prone and do not contain any high-level information related to diagnosis. In recent years, deep learning-based models have achieved state-of-the-art results in feature representation learning. Zhu et al. [8] proposed a deep learning-based survival model that takes 1024×1024 image patches as input and treats the classification outputs as the patient risk scores for survival analysis. However, in practice, pathologists or doctors make diagnostic decisions based on cellular level image information, and these discriminative details could be lost in Zhu’s architecture, which downsamples the inputs with size 1024×1024 to the last convolutional feature maps with size 20×20. More importantly, this system misses the opportunity to use the well-developed survival analysis methods, such as the Cox proportional hazards model and its variants [31, 36, 37], to identify and interpret biomarkers. So far, those works that can combine the deep learning framework and the classic survival analysis methods remain absent. Finally, individual cellular features are currently aggregated into a patient level feature vector using relatively simple statistically based methods such as taking the mean, median or standard variation of each feature dimension [1, 6, 7]. More advanced local feature aggregation methods, such as bag of words (BoW) [38] or sparsity-based BoW variations [39, 40], are not investigated.

**Fig. 1:**
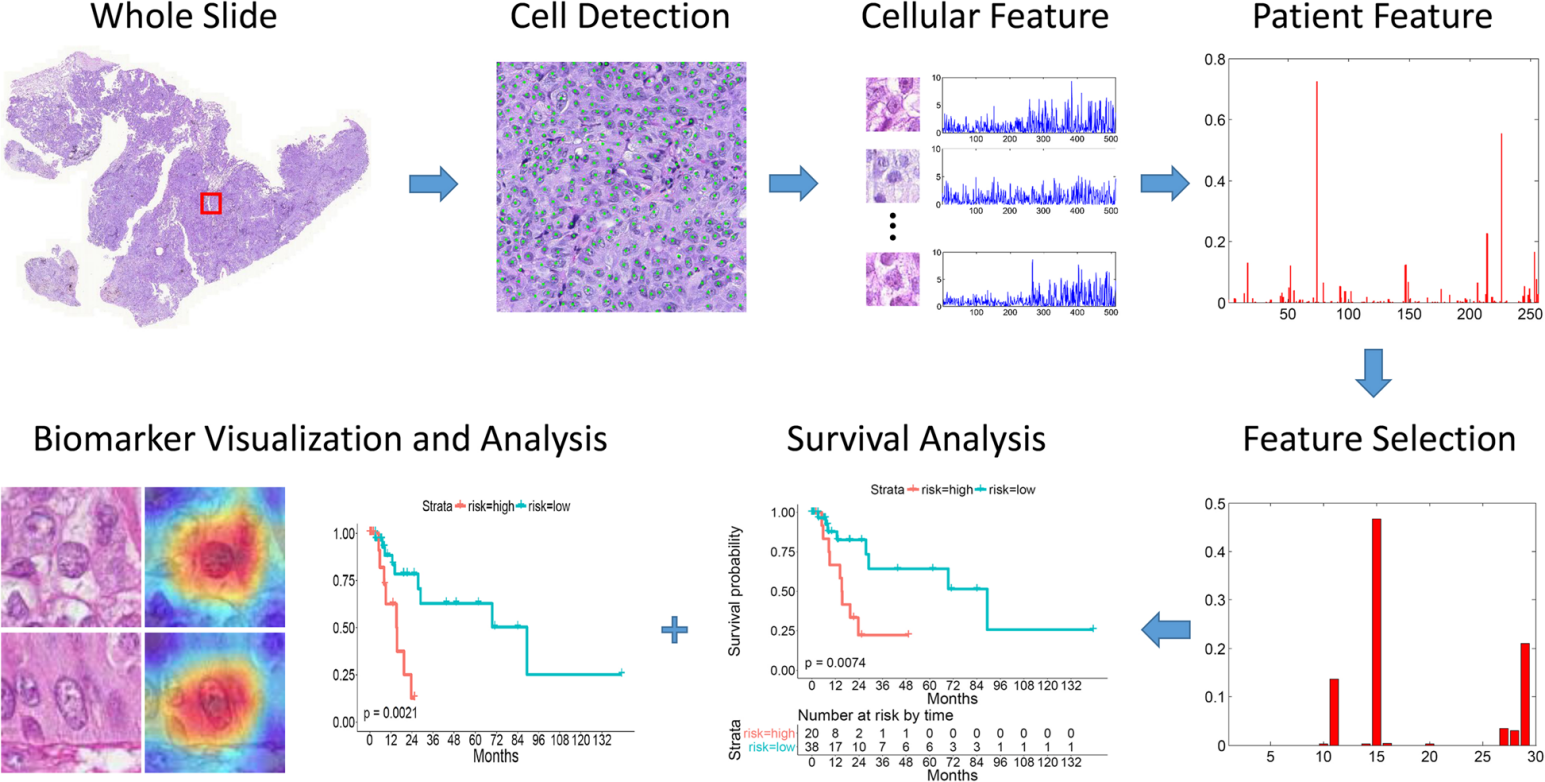
An overview of the proposed system for lung cancer survival analysis

To overcome the aforementioned challenges, we propose a survival analysis system that takes advantage of the emerging deep learning framework [20, 21] and well-studied survival analysis methods [31, 36, 37]. An overview of the proposed system is provided in Fig. 1, which consists of three main components: 1) An end-to-end cellular feature learning module using a deep neural network with global average pooling. The learned cellular representations encode high-level biologically relevant information without the requirement of individual cell segmentation, and then are aggregated into patient-level feature vectors by using a locality-constrained linear coding (LLC)-based bag of words (BoW) encoding algorithm. 2) A Cox proportional hazards model with an elastic net penalty for robust feature selection and survival analysis. 3) A biomarker interpretation module which can help localize the image regions that contribute to the survival model’s decisions. Extensive experiments demonstrate that the proposed survival model provides excellent predictive power for testing data in terms of two commonly used survival analysis metrics: the log-rank test (*p*-value) of the Kaplan-Meier estimate and the concordance index (*c*-index). Furthermore, the proposed system can easily visualize the selected biomarkers, which can serve as regions of interest (ROIs). In this scenario, pathologists or doctors can validate the automated generated survival analysis results by examining these ROIs using raw image data. We argue that this system should receive greater future investment.

## Methods

### Cell detection via end-to-end learning

In pathological image analysis, cells are the regions of interest, and it is important to achieve accurate and robust cell detection. Many conventional CNN architectures [9–12] usually adopt the sliding window strategy to make dense predictions in the testing stage, which is computationally expensive. Fortunately, an FCN [30] architecture is proposed to upsample the output layer to a higher resolution which is the same as the input image dimension. Furthermore, in order to propagate more contextual information to higher resolution layers, Ronneberger et al. have proposed a U-Net architecture [41] which makes the expansive and contracting paths more or less symmetrical via gradually upsampling layers in the expansive path and concatenating them with corresponding layers in the contracting path. The U-Net architecture has been proven to be effective for cell segmentation and tracking [42]. In this work, we adopt the U-Net architecture for cancer cell detection.

Denote $\mathcal {T} = \{(x,y) \in \mathcal {X} \times \mathcal {Y}\}$ as the training data, where *x* represents a training image and *y*=*v*∗*G* is the corresponding cell center probability map, where *v*(*i*,*j*)=1 if there is a human cell center annotation at pixel location (*i*,*j*), and otherwise *v*(*i*,*j*)=0. *G* denotes a Gaussian kernel with standard deviation *σ*. Let *o* denote the output, and then the loss function can be defined as:

1 $$ \mathcal{L}(y,o)= \frac{1}{2}\sum_{i=1}^{h}\sum_{j=1}^{w}(y^{ij} + \beta \bar{y})(y^{ij} - o^{ij})^{2},   $$

where *h* and *w* denote the height and width of *x*, respectively, and $\bar {y}$ represents the mean value of *y*. *β* is a predefined constant which is chosen as *β*=0.2 in our implementation. The final cell center coordinates are achieved via non-maximum suppression on the output map *o*. Please refer to [42] for more details of the U-Net architecture.

### Biological information bearing feature learning

#### Cellular feature extraction

After cell localization, discriminative cell representations must be learned for survival analysis. In the current literature, cellular descriptors are still composed using hand-crafted image features, and a proportion of them, such as cell size and shape features, rely on cell segmentation. Inaccurate cell segmentation will reduce the discriminative power of these features. Recently, image features based on the outputs of the last fully connected layers of CNN models have emerged as state-of-the-art generic representations for visual recognition [43–46]. Inspired by the success of CNN features, in this work, we propose to train a deep neural network to classify cells into different risk groups based on the corresponding patient survival times, and we build cell descriptors using activations within the learned deep neural network.

**Fig. 2:**
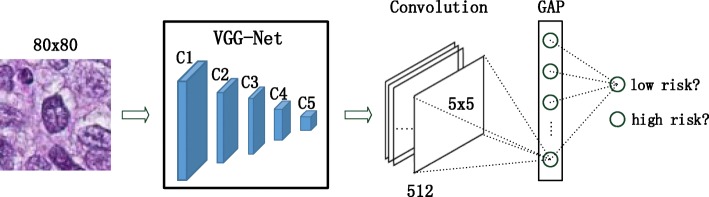
An overview of the proposed cell feature learning framework. The activation values of the global average pooling (GAP) layer are used as the cell representation

The proposed architecture in Fig. 2 is based on the VGG-Net [47], from which we remove the layers after *conv*5 and instead add a convolutional layer followed by a global average pooling (GAP) layer with pooling size 5 and stride 5, which is then directly fed into the softmax layer for outputting the risk scores. The newly added convolutional layer selects 3×3 kernels with 512 feature maps as well as stride size equal to 1 and pad size of 1. The activations of the GAP layer are used as feature representations of cells for the subsequent survival analysis. The GAP layer design is inspired by [48], which uses a GAP layer as a structural regularizer to help the deep neural network prevent overfitting and improve the generalization ability. Later, it is used to localize the discriminative image regions (attention) in [46] for solving classification tasks. Note that the global average pooling summarizes the spatial information, and thus it is inherently robust with respect to spatial translation of the inputs. This property is essentially important for robust cell feature learning because inaccurate or inconsistent cell center localizations will directly lead to input translations.

**Fig. 3:**
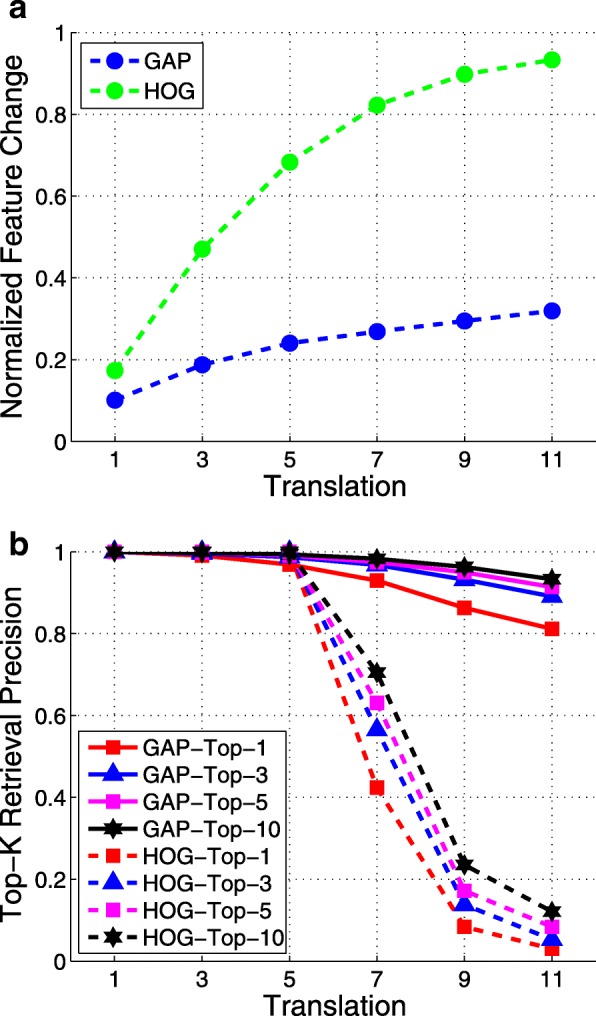
Robustness analysis of cell feature representation versus simulated image translations via randomly shifting cell centers in four directions by 1, 3, 5, 7, 9, and 11 pixels, respectively. Left: Feature variation versus different image translations; Right: Cell retrieval accuracy versus different image translations

To illustrate the robustness of the GAP layer with respect to translations of the inputs, we provide both quantitative and qualitative analyses as follows. Specifically, we first randomly sample 1000 cells from histopathological images of our dataset, The Cancer Genome Atlas (TCGA), pass them forward through the trained network, and treat the activations of the GAP layer as their features $\mathbf {X} \in \mathbb {R}^{D \times N}$, where *D*=512 and *N*=1000. Then, we randomly shift the cell center coordinates in four directions (left, right, top, and bottom) by a certain amount *t*, and their corresponding features $\mathbf {X}_{t} \in \mathbb {R}^{D \times N}$ are extracted as well. The Euclidean distances between the features of translated cells and the originals, $\mathbf {D}_{t} = \Vert \mathbf {X} - \mathbf {X}_{t} \Vert _{2} \in \mathbb {R}^{N}$, are computed. We repeat this process for different translation amounts, with *t*={1,3,5,7,9,11} pixels. For comparison, the histograms of oriented gradients (HOG) [49] features are also computed. The medium values of **D**_*t*_ normalized by the medium values of the inter-cell distances of **X** against different *t* are plotted in Fig. 3a. It can be observed that, compared with the HOG image features, the perturbations of GAP features caused by input translations are smooth and insignificant. Furthermore, for each translated cell, we conduct top-k retrieval against the original cells and compute the precision. The top-k retrieval precision values for different *t* are shown in Fig. 3b, showing that the retrieval accuracy using GAP features remains at a high value even with a large translation (for example, 11 pixels). In contrast, the retrieval performance using HOG features deteriorates significantly as the translation grows. The robustness of the GAP cellular features with respect to input translations will effectively compensate for the inaccuracy or inconsistency of cell detection.

**Fig. 4:**
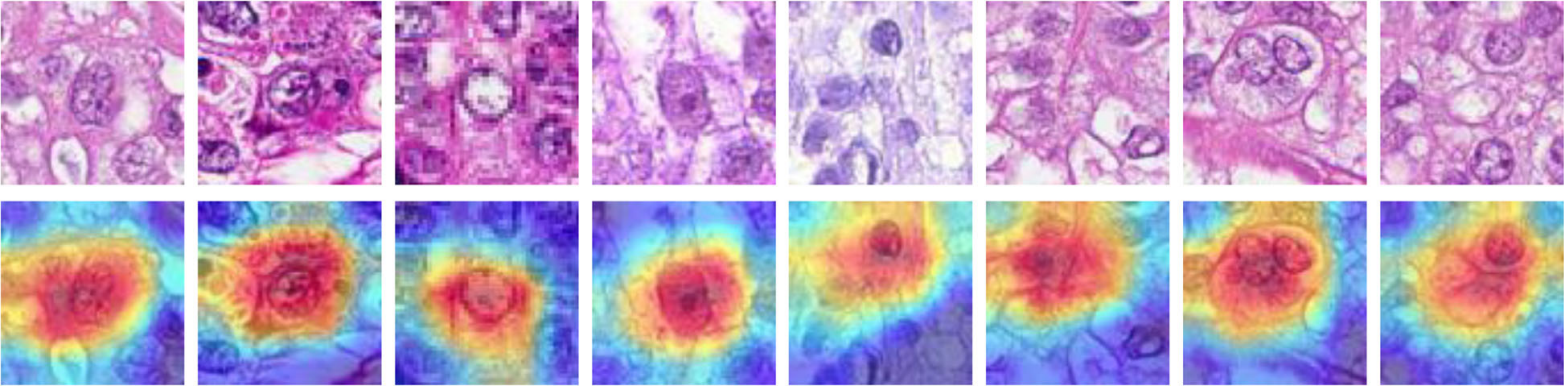
Examples of the generated CAMs for several training patches. The maps in the second row highlight the discriminative image regions for cell classification. Please refer to [46] for the details of computing CAMs

In addition, we probe into the neural network and localize the discriminative image regions (*attention*s) for cell classification by following the method in [46]. Several examples of the training patches and the computed class activation maps (CAMs) are shown in Fig. 4. It can be observed that the network generally places greater *attention*s on the central regions which are desired. However, when the cells are not localized in the image patch centers due to inaccurate cell detection, the network will adjust its *attention*s accordingly. This further shows that the activations of the GAP layer are robust with respect to image translations and are suitable for cell representations.

#### Aggregating cellular features

Given a patient *p* and a set of *D*-dimensional cellular descriptors $\mathbf {X} = [\mathbf {x}_{1},\mathbf {x}_{2},...,\mathbf {x}_{N}] \in \mathbb {R}^{D \times N}$ from *p*, we aim to aggregate individual cellular representations **X** into a single feature vector, **f**, as *p*’s representation. One of the simplest yet most effective local descriptor aggregation methods is the BoW model [38]. Given a learned codebook with *M* entries, $\mathbf {B} = [\mathbf {b}_{1},\mathbf {b}_{2},...,\mathbf {b}_{M}] \in \mathbb {R}^{D \times M}$, the BoW method converts each cellular descriptor **x**_*i*_ into an *M*-dimensional code $\mathbf {c}_{i} \in \mathbb {R}^{M}$, and then all of the cellular codes $\mathbf {C}= [\mathbf {c}_{1},\mathbf {c}_{2},...,\mathbf {c}_{N}] \in \mathbb {R}^{M \times N}$ are pooled into a single vector, $\mathbf {f} \in \mathbb {R}^{M}$. There are many encoding methods, such as hard vector quantization (VQ) [50] and sparse coding-based soft VQ [39, 40]. Among these works, LLC [40] is used to project each descriptor into its local coordinate system for patientwise representation learning. Letting $\mathbf {B}_{i} \in \mathbb {R}^{D \times k}$ denote the *k*-nearest neighbors of **x**_*i*_, where *k*<<*M*, the code **c**_*i*_ can be achieved by solving a small linear system:

2 $$ \min_{\mathbf{C}} \sum_{i=1}^{N} \Vert \mathbf{x}_{i} - \mathbf{c}_{i}\mathbf{B}_{i} \Vert^{2}, \;\;\; st. \; \mathbf{1}^{T}\mathbf{c}_{i} = 1, \: \forall{i}   $$

The final feature representation **f** for patient *p* can be achieved via sum pooling or max pooling, followed by *ℓ*_2_ normalization, **f**=**f**/∥**f**∥_2_. The poolings are computed as: 1) sum pooling: $\mathbf {f} = \sum _{i=1}^{N} \mathbf {c}_{i}$; 2) max pooling: **f**= max(**c**_1_,...,**c**_*N*_).

### Survival analysis

Given a set of observations (**f**_*i*_,*y*_*i*_,*δ*_*i*_),*i*=1,2,...,*P*, where *P* is the number of observations, *y*_*i*_ is the observed time to the event of interest for individual *i*, *δ*_*i*_=1 if an event occurred at that time and 0 if the observation has been censored, and $\mathbf {f}_{i} = [\mathbf {f}_{i1}, \mathbf {f}_{i2},...,\mathbf {f}_{iM}] \in \mathbb {R}^{M}$ is the set of features or predictor variables obtained at time 0. The objective of survival analysis is to reveal the relationship between predictor variables, such as the image feature vector **f** in this work, and *survival time*. In health informatics, the Cox proportional hazards model [31] is one of the most commonly used approaches for survival time analysis and is defined as:

3 $$ s_{i}(t) = s_{0}(t)e^{\mathbf{f}_{i}^{T} \beta},   $$

where *s*_*i*_(*t*) is the hazard for observation *i* at time *t*, *s*_0_(*t*) is the baseline hazard and is left unspecified, and $\beta \in \mathbb {R}^{M}$ is the parameter vector. The estimation of *β* is obtained by maximizing the partial log likelihood:

4 $$ L(\beta) = \prod_{i:\delta_{i}=1}^{P} \frac{e^{\mathbf{f}_{i}^{T} \beta}}{\sum_{j \in R_{i}} e^{\mathbf{f}_{j}^{T} \beta}},   $$

where *R*_*i*_={*j*|*y*_*j*_≥*t*_*i*_}. For high-dimensional data, with *M*>*P*, the *ℓ*_1_ penalty (lasso) [51] or *ℓ*_2_ (ridge regression) is added to avoid degenerated solutions.

The *ℓ*_1_ penalty tends to generate sparse solutions, which are often desired for feature selection. However, as indicated in [36], this can also cause one significant problem: if the two predictors are strongly correlated, the lasso will pick one and entirely ignore the other. To tackle this problem, we add the elastic net penalty [37], which is a mixture of *ℓ*_1_ and *ℓ*_2_, to (4): the problem thus becomes:

5 $$ L_{net}(\beta) = L(\beta) - \lambda(\alpha \Vert \beta \Vert_{1} + \frac{1}{2}(1 - \alpha) \Vert \beta \Vert_{2}^{2}),   $$

where *L*(*β*) is defined in Eq. (4), ∥·∥_1_ denotes the *ℓ*_1_ penalty, $\Vert \cdot \Vert _{2}^{2}$ denotes the *ℓ*_2_ penalty, and *α* is used to balance between *ℓ*_1_ and *ℓ*_2_. In our implementation, we solve (5) using the cyclical coordinate descent algorithm [36], and its implementation can be found in the R package **glmnet**^3^.

### Evaluation

We use two metrics to measure the predictive power of the survival model: the Kaplan-Meier estimate (KME) [32] that can effectively measure the survival differences between two or more groups, and the concordance index (*c*-index) [52] that can reveal the relative risks between patients.

**Kaplan-Meier Estimate**. In clinical trials, it is important to be able to accurately and robustly measure the fraction of patients who survive after a certain amount of time after treatment in spite of censored observations. For this purpose, the Kaplan-Meier estimate is the simplest yet most effective way to compute the survival rates. For two or more groups of subjects, the log-rank test is conducted to measure the significant difference between their survival distributions. The survival outcomes of two groups are considered as significantly different if the *p*-value of the log-rank test is less than 0.05.

**Concordance Index**. This is another commonly used metric to measure survival model performance, which is calculated as the fraction of all pairs whose predicted survival risks are correctly ordered among all subjects that can actually be ordered. The survival time *t*_*i*_/ *t*_*j*_ for patient *p*_*i*_/ *p*_*j*_ can be ordered if *p*_*i*_ is uncensored and *t*_*i*_<*t*_*j*_. Note that *t*_*j*_ would be the censoring time if *p*_*j*_ is censored. Let *G*=(*V*,*E*) denote a directed graph, where the vertices *V* denote all of the patients, and a directed edge *e*_*ij*_ exists between two nodes, *v*_*i*_ and *v*_*j*_, if the corresponding patient *p*_*i*_ of *v*_*i*_ is uncensored and *t*_*i*_<*t*_*j*_. The edges can only originate from those uncensored nodes. Given a pair of patients (*p*_*i*_,*p*_*j*_)∈*E* and their risk scores *r*_*i*_ and *r*_*j*_,*p*_*i*_ and *p*_*j*_ are considered concordant if *r*_*i*_>*r*_*j*_. For the concordance index (*c*-index), the value of 0.5 is a random guess, and 1 is the best.

## Experimental results

### Dataset

The proposed framework is validated using a dataset downloaded from TCGA data portal^4^. TCGA is a collection of cancer specimens with additional clinical information and histopathology slide images. In total, 121 patients with image annotation and entire survival information are collected, and they are randomly partitioned into two sets, Set I and Set II, which contain 63 and 58 patients, respectively. The detailed description of the two sets is summarized in Table 1. A patient is labeled as high risk if his/her known days to death value is less than *T*_1_, and low risk if either the days to death or days to last follow up value is greater than *T*_2_. Note that those patients whose days to death values are unknown and whose days to last follow up values are less than *T*_1_ are not included for cell feature training. For example, when *T*_1_=*T*_2_=3 years, among the 63 patients in Set I, there are 14/12 patients in the low/high risk group; among the 58 patients in Set II, there are 8 with low risk and 15 with high risk. For each patient, an image patch with size 2356×1304 is sampled from the cancerous regions of the histopathology slide image under 20X magnification for image feature extraction.

**Table 1:** Dataset description

Dataset	Description
Set I	63 patients, 14 in low risk group, 12 in high risk group
Set II	58 patients, 8 in low risk group, 15 in high risk group

### Pre-processing

#### Cell detection

**Fig. 5:**
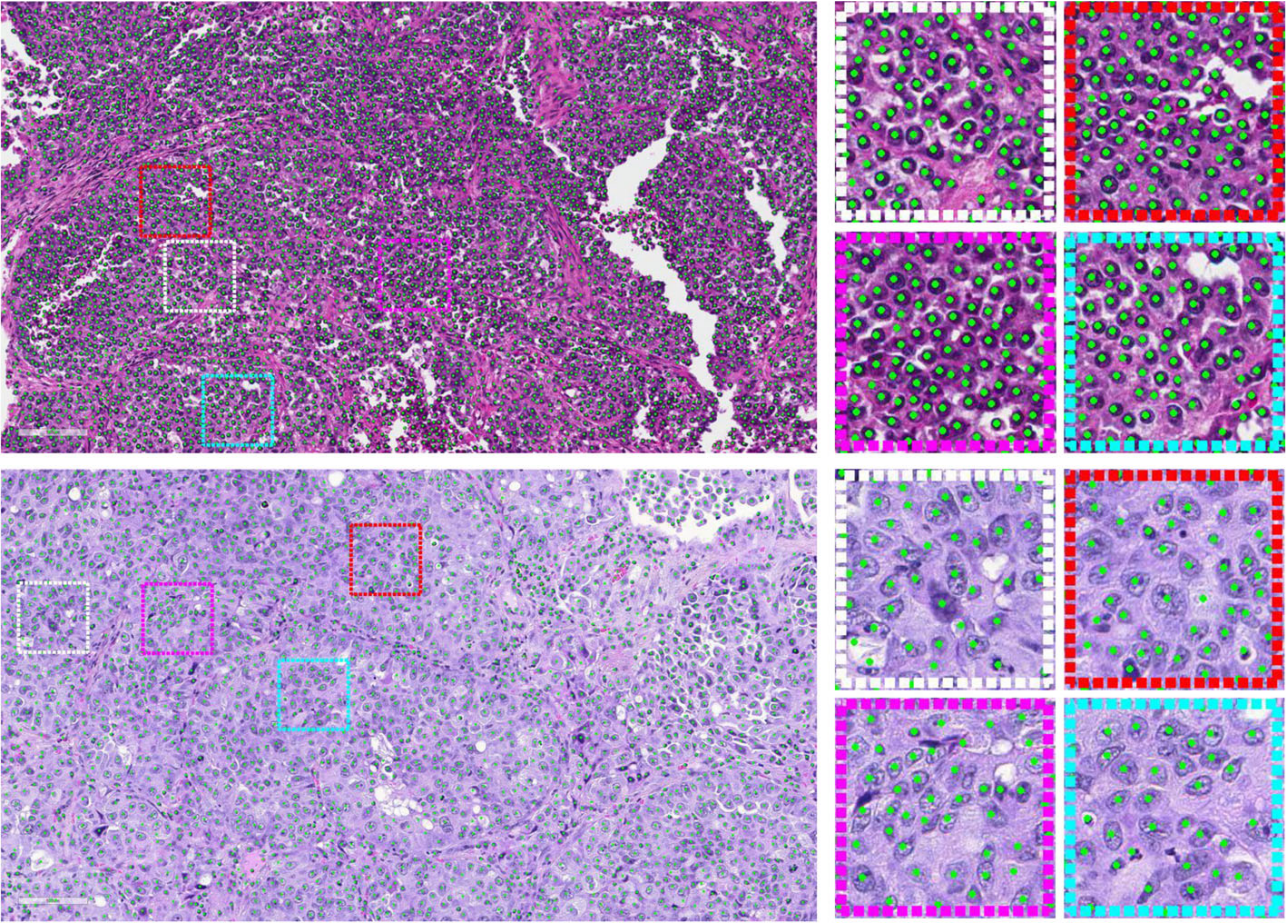
Qualitative cell detection results for two large image patches. Several zoomed-in patches are shown on the right part. In the upper row, 6087 cells are detected, and 2631 cells are detected in the lower row

In this work, we implemented the U-Net architecture using Theano^5^ and Keras^6^. Both training and testing are conducted on a machine equipped with an Intel Xeon E5-1650 CPU and an NVIDIA Quadro K4000 GPU. The qualitative cell detection results for two large image patches are shown in Fig. 5, and several zoomed-in patches are also provided for better illustration. It may be observed that the trained model can provide desirable cell center localization results.

#### Feature learning

The feature learning model is fine-tuned based on the pretrained VGG model^7^ using Caffe [53] implementation on an NVIDIA Quadro K4000 GPU. For each training image, cell centers are localized using the aforementioned cell detection method. For each detected cell, an 80×80 patch around it is cropped as a training sample, and the label of the training sample is decided by the corresponding patient’s risk status. For each patient, 2000 cells are randomly selected for feature extraction. The model is trained using a stochastic gradient descent algorithm with the initial learning rate set as 0.00001 and the mini-batch size of 10. The training is stopped after 100,000 iterations.

### Survival analysis without feature selection

We validate the proposed cell feature learning framework for survival analysis using two different setups: 1) Evaluation I, using the selected 26 patients in Set I as training and the rest of Set I plus all patients in Set II as testing; 2) Evaluation II, using the selected 23 patients in Set II as training and the remaining patients in Set II plus all patients in Set I as testing. In both experimental setups, we maximize the testing set sizes.

For the training set, a binary classifier (logistic regression used in this paper) is trained to classify each patient as low risk or high risk. For the testing set, the model output *r*_*i*_∈[0,1] for the patient *p*_*i*_ is treated as his/her survival risk score, with a larger value denoting a higher survival risk and vice versa. When computing *p*-values, a risk score threshold *r*=0.5 is used to partition the testing patients into two groups, *low*/*high* risk, by their predicted *r*-values and then to compute the *p*-values from the log-rank test. When computing *c*-index values, the raw risk score *r* is used, and no binary thresholding is involved.

The detailed survival analysis results of Evaluation I and Evaluation II using both *p*-value and *c*-index metrics are listed in Table 2. Note that in addition to the patient features aggregated via LLC reconstruction, the conventional encoding method, BoW, is also tested. For both BoW and LLC encoding, the dictionary **B** is learned via *k*-means clustering with *k* equal to 256. Meanwhile, two other local feature aggregation methods are also tested for comparison:

**Table 2:** Survival analysis results with different feature encoding algorithms

Method	Evaluation I		Evaluation II	
	*p*-value	*c*-index	*p*-value	*c*-index
Cellular voting	0.0290	0.5103	0.0320	0.5739
Aggregate statistic [6, 7]	0.0083	0.6798	0.2200	0.6750
BoW encoding	0.0290	0.6591	**0.0045**	**0.6770**
LLC-sum encoding	0.0110	0.6874	0.0260	0.6751
LLC-max encoding	**0.0031**	**0.6911**	0.0470	0.6770

Note that lower *p*-values are better, whereas higher *c*-index values are better

1. Cellular Voting. Here, we treat the softmax output of the deep learning model (see Fig. 2) as the cell’s risk score, and the patient’s risk score is determined by averaging all of the cellular scores.
2. Aggregate Statistic. As in [1, 6, 7], the mean, median, and standard variation of each cellular feature are computed and then concatenated into one single vector. The resulting feature dimension is 1536.

From Table 2, we observe the following: 1) In terms of both *p*-value and *c*-index metrics, the built survival model achieves satisfactory survival prediction outcomes with varying cellular feature aggregation methods. For example, the *p*-values of the survival models using BoW, LLC-sum and LLC-max encoding methods are all less than 0.05 under both setups. Their *c*-index values are close to 0.7. As a reference, a *c*-index value of 0.629 is achieved in [8] on a different dataset, the National Lung Screening Trial (NLST) lung cancer data. We can conclude that the learned cellular features indeed encode patient survival information and effectively generalize the testing data. 2) Other feature encoding methods such as cellular voting and aggregate statistics are not able to produce robust and accurate survival predictions for both sets of testing data. For example, though the cellular voting method exhibits good *p*-value performance, the *c*-index value is not satisfactory; in addition, the aggregate statistic method achieves good results in terms of *p*-value and *c*-index in Evaluation I, but the *p*-value performance in Evaluation II is poor.

**Fig. 6:**
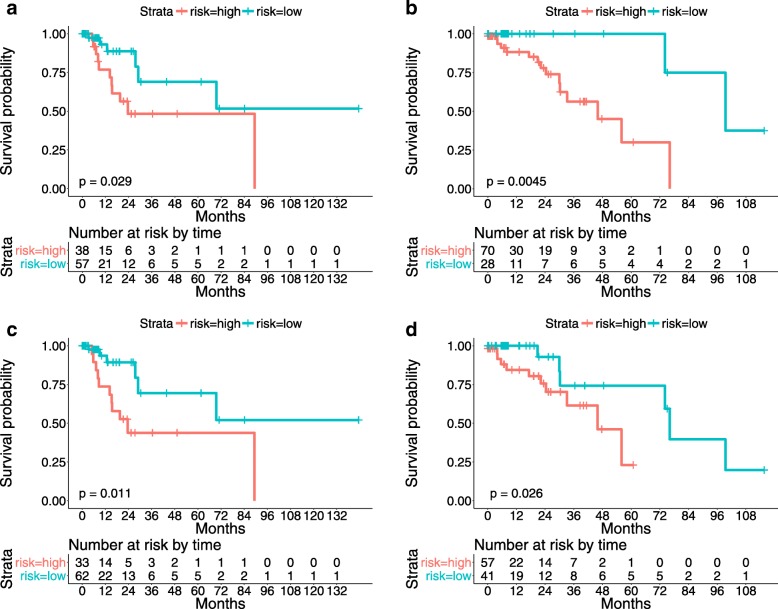
The proposed algorithm predicts the survival outcomes of testing patients. **a** and **b**: Kaplan-Meier curves of lung cancer patients stratified using the proposed survival model with BoW encoding on the testing data of Evaluations I and II, respectively; **c** and **d**: Kaplan-Meier curves of lung cancer patients stratified using the proposed survival model with LLC-sum encoding on the testing data of Evaluations I and II, respectively

The Kaplan-Meier curves stratified using the proposed survival model are shown in Fig. 6. It can be observed that the numbers of the patients who are at risk at the beginning of each time interval are also reported. These table values can be used to reconstruct the Kaplan-Meier curves and provide us with better insight into the survival prediction results.

### Survival analysis with feature selection

We conduct survival analysis with feature selection under two different setups: 1) Evaluation I, which uses all patients in Set I for training and all patients in Set II for testing; 2) Evaluation II, which uses all patients in Set II for training and all patients in Set I for testing.

**Fig. 7:**
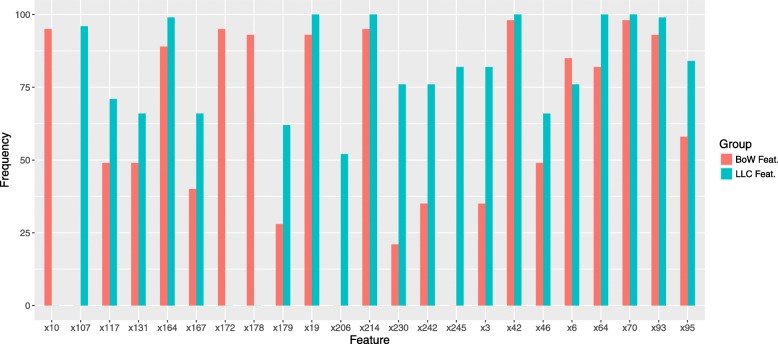
The frequencies of the selected features for both BoW and LLC-sum encoding

Feature selection is performed by solving Eq. (5), with *α* set as 0.2 in all experiments. We repeat this procedure 100 times on the training data with 10-fold cross validation and record the frequencies of the chosen features. For all encoding methods, the top 30 selected features are chosen for fair comparison. For both BoW and LLC-sum encoding under Evaluation I, several sample selected features and their frequencies are shown in Fig. 7. Since BoW and LLC-sum features are derived from the same dictionary **B**, their selected features can be aligned for comparison. It can be observed that the selected features, which correspond to certain entries (or cluster centers) in the dictionary **B**, are very consistent for both encoding methods. We can conclude that the cells that fall into those centers carry discriminative information about patient survival outcomes.

**Fig. 8:**
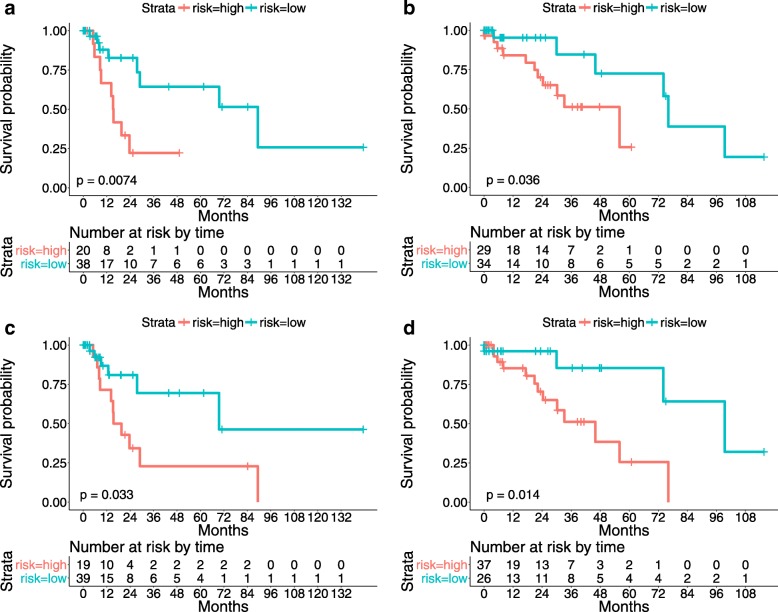
The proposed algorithm predicts the survival outcomes of testing patients using selected features. **a** and **b**: Kaplan-Meier curves of lung cancer patients stratified using the proposed survival model with LLC-sum encoding on the testing data of Evaluations I and II, respectively; **c** and **d**: Kaplan-Meier curves of lung cancer patients stratified using the proposed survival model with LLC-max encoding on the testing data of Evaluations I and II, respectively

After feature selection, a survival model is built on the training data and its predictive power is validated with the testing data. The Kaplan-Meier curves and the *p*-values of the log-rank test using both LLC-sum and LLC-max encodings are provided in Fig. 8. In addition, the detailed numerical analysis results are listed in Table 3. Note that 1) The reported table values denote average results of the test data of both Evaluations I and II; 2) The survival prediction outcomes without feature selection are also computed for comparison. It can be observed that the adopted feature selection strategy which adds an elastic net penalty to the Cox model (Eq. 5) can find features that are highly correlated with patient survival outcomes. For example, for both LLC-sum and LLC-max encodings, the selected 30 features achieve similar performance as using all features (256 dimensions).

**Table 3:** Comparison of survival models using different aggregating algorithms

Method	*p*-value		*c*-index	
	All	Top 30	All	Top 30
Aggregate Statistic [6, 7]	0.1247	0.1344	0.6564	0.6633
BoW Encoding	0.0072	0.1190	0.6678	0.6200
LLC-sum Encoding	0.0204	0.0217	0.6713	0.6449
LLC-max Encoding	0.0298	0.0235	0.6665	0.6767

### Biomarker analysis and visualization

In this experiment, we use all patients in Set I for training and all patients in Set II for testing. No cross validation is involved.

**Fig. 9:**
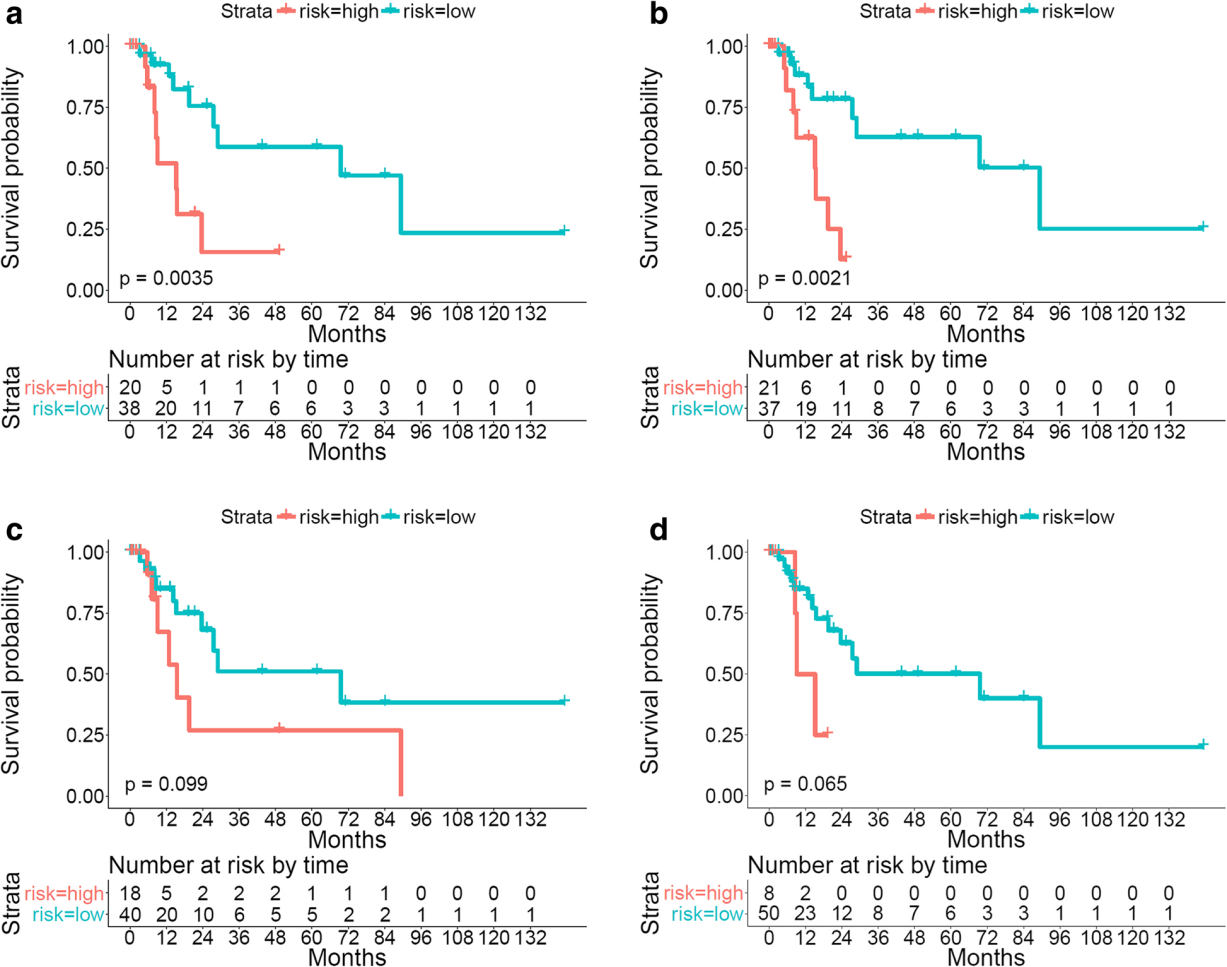
Univariate survival analysis using selected features

We first conduct univariate survival analysis using the selected features. Four features, x70, x93, x107 and x164, are chosen for examination. For the *i*-th feature, $\mathbf {X}_{i} \in \mathbb {R}^{P}$ denotes the feature values for all patients, where *P* is the number of patients. A stump classifier is then trained on the training patients, and the learned threshold is used to partition the testing patients into the *low-* and *high-*risk groups. When computing the *c*-index, no risk scores need to be learned, and the raw feature values are directly used for calculation for both training and testing set. The survival analysis results of both training and testing data are provided in Table 4, and the Kaplan-Meier curves are provided in Fig. 9. It can be observed that some biomarkers do carry discriminative survival information. For example, x93 produces excellent prediction results in terms of both *p*-value and *c*-index on both training and testing sets. In fact, under the same setup, x93 achieves a better *c*-index value, 0.7157, than models built using all (0.6834) and the top 30 selected features (0.6750).

**Table 4:** Survival analysis performance of discovered biomarkers

Feature ID	*p*-value		*c*-index	
	Train	Test	Train	Test
x70	0.0001	0.0035	0.7530	0.3683
x93	0.0059	0.0021	0.7382	0.7157
x107	0.0460	0.0990	0.6024	0.5448
x164	0.0099	0.0650	0.6395	0.6428

**Fig. 10:**
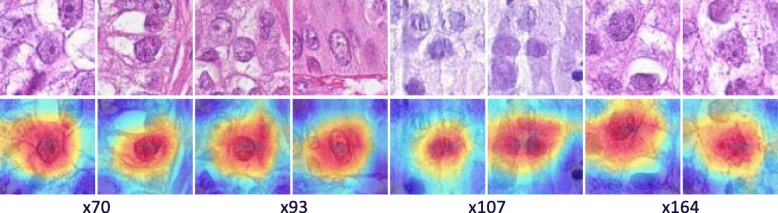
Visualization of discovered biomarker samples and the generated CAMs

**Fig. 11:**
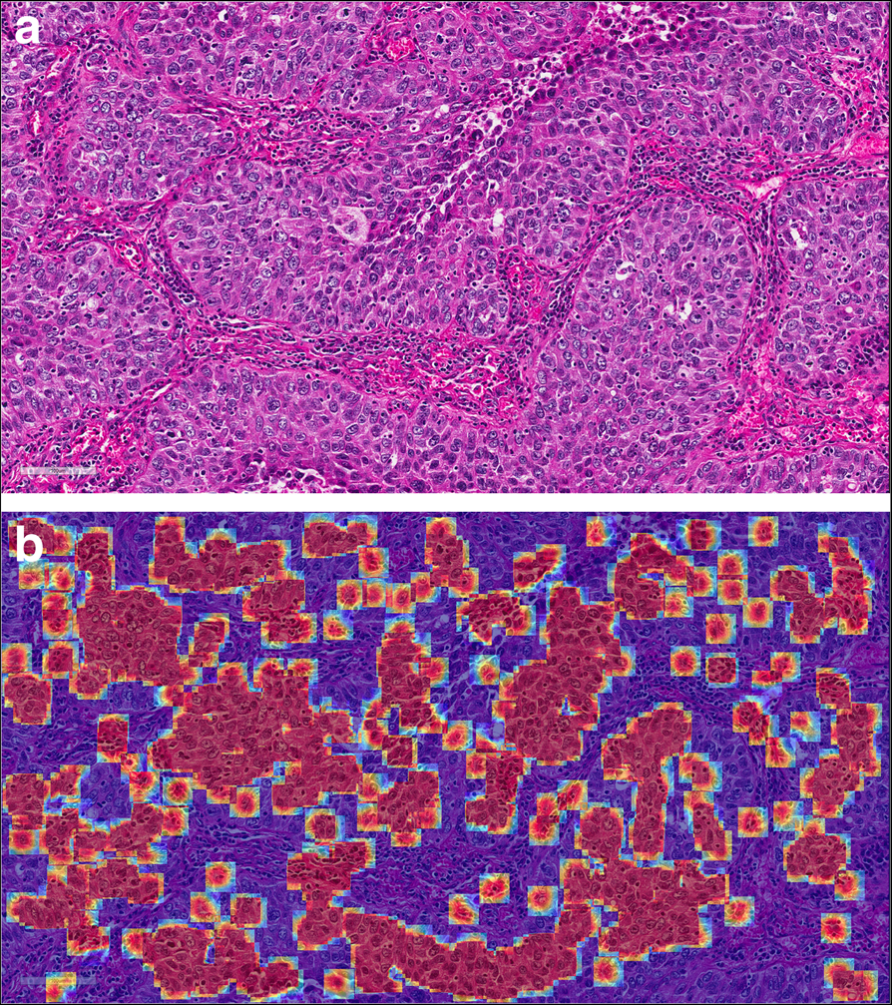
Left: An example image patch; Right: The cells that contribute to the survival model’s decision are highlighted

Since the feature value is closely related to the number of occurrences of cells belonging to a certain cell cluster, it will be helpful if those cells can be visualized. Note that the cluster center dictionary **B** is learned via *k*-means clustering, which means that the cell samples that fall into a certain cluster can be efficiently identified. Several cell samples corresponding to features x70, x93, x107, and x164 are shown in Fig. 10. Note that the survival model makes predictions using only the cells that fall into the selected cluster centers, and thus it would be helpful if these *attention* cells can be visualized. One example can be found in Fig. 11, which shows that the cells which contributed to the survival model’s decision are localized and that they are nearly all tumor cells. This suggests that our survival analysis system can not only provide numeric conclusions regarding patients’ survival outcomes but can also provide visual evidence supporting the decisions. We argue that the latter ability is also important, since it allows the pathologists or doctors the opportunity to re-assess those biomarkers using their expertise and knowledge. For the computer-aided diagnosis system, we believe that this machine learning framework should receive greater investment in the future.

## Conclusion

In this work, we have proposed a segmentation-free survival analysis system that takes advantage of the recently emerging deep learning framework and well-studied survival analysis methods such as the Cox proportional hazards model. Extensive experiments demonstrate that the proposed survival model offers excellent predictive power for the TCGA lung cancer dataset in terms of two commonly used survival analysis metrics: the log-rank test (*p*-value) of the Kaplan-Meier estimate and concordance index (*c*-index). In addition, we provide an approach to visualize the discovered biomarkers, which can serve as concrete evidence supporting the survival model’s decisions.

## Data Availability

The TCGA data that support the findings of this study are available from https://tcga-data.nci.nih.gov/docs/publications/tcga/, which is a collection of cancer specimens, with additional clinical information and histopathology slide images. The code of this paper is available now at https://github.com/chrisleiNWU/Lung-Cancer-Surviarl-Analysis.

## Abbreviations

AC: AdenoCarcinoma
BoW: Bag of words
CAM: Class activation maps
CNNs: Convolutional neural networks
FCN: Fully convolutional network
FRST: Fast radial symmetry transform
GAP: Global averaging pooling
HOG: Histogram of oriented gradients
KME: Kaplan-Meier estimate
LLC: Locality-constrained linear coding
LoG: Laplacian of Gaussian
NSCLC: Non-small cell lung cancer
ROIs: Regions of interest
SC: Squamous cell carcinoma
SCLC: Small cell lung cancer
TCGA: The cancer genome Atlas
VQ: Vector quantization

## Footnotes

1 http://www.lung.org/lung-health-and-diseases/lung-disease-lookup/lung-cancer/resource-library/lung-cancer-fact-sheet.html

2 https://www.cancer.org/cancer/non-small-cell-lung-cancer/about/what-is-non-small-cell-lung-cancer.html

3 http://web.stanford.edu/~hastie/glmnet/glmnet_alpha.html

4 https://tcga-data.nci.nih.gov/docs/publications/tcga/

5 http://deeplearning.net/software/theano/

6 https://github.com/fchollet/keras

7 https://github.com/BVLC/caffe/wiki/Model-Zoo

## References

[1] Yu K-H, Zhang C, Berry GJ, Altman RB, Ré C, Rubin DL, Snyder M. Predicting non-small cell lung cancer prognosis by fully automated microscopic pathology image features. Nat Commun. 2016; 7. 10.1038/ncomms12474. 10.1038/ncomms12474 PMC4990706 27527408

[2] Xing F, Yang L (2016). Robust nucleus/cell detection and segmentation in digital pathology and microscopy images: A comprehensive review. IEEE Rev Biomed Eng.

[3] Beck AH, Sangoi AR, Leung S, Marinelli RJ, Nielsen TO, van de Vijver MJ, West RB, van de Rijn M, Koller D (2011). Systematic analysis of breast cancer morphology uncovers stromal features associated with survival. Sci Transl Med.

[4] Xing F, Su H, Neltner J, Yang L (2014). Automatic ki-67 counting using robust cell detection and online dictionary learning. IEEE Trans Biomed Eng.

[5] Sertel O, Kong J, Catalyurek UV, Lozanski G, Saltz JH, Gurcan MN (2009). Histopathological image analysis using model-based intermediate representations and color texture: Follicular lymphoma grading. J Sig Process Syst.

[6] Wang H, Xing F, Su H, Stromberg A, Yang L (2014). Novel image markers for non-small cell lung cancer classification and survival prediction. BMC Bioinformatics.

[7] Yao Jiawen, Wang Sheng, Zhu Xinliang, Huang Junzhou (2016). Imaging Biomarker Discovery for Lung Cancer Survival Prediction. Medical Image Computing and Computer-Assisted Intervention – MICCAI 2016.

[8] Zhu X, Yao J, Huang J. Deep convolutional neural network for survival analysis with pathological images. In: 2016 IEEE International Conference on Bioinformatics and Biomedicine (BIBM): 2016. p. 544–7. 10.1109/BIBM.2016.7822579.

[9] LeCun Y, Bottou L, Bengio Y, Haffner P (1998). Gradient-based learning applied to document recognition. Proc IEEE.

[10] LeCun Y, Kavukcuoglu K, Farabet C. Convolutional networks and applications in vision. In: Circuits and Systems (ISCAS), Proceedings of 2010 IEEE International Symposium On. IEEE: 2010. p. 253–6. 10.1109/iscas.2010.5537907.

[11] Deng L, Yu D (2014). Deep learning: methods and applications. Found Trends Sig Process.

[12] LeCun Y, Bengio Y, Hinton G (2015). Deep learning. Nature.

[13] Parvin B, Yang Q, Han J, Chang H, Rydberg B, Barcellos-Hoff MH (2007). Iterative voting for inference of structural saliency and characterization of subcellular events. TIP.

[14] Byun J, Verardo MR, Sumengen B, Lewis GP, Manjunath B, Fisher SK (2006). Automated tool for the detection of cell nuclei in digital microscopic images: application to retinal images. Mol Vis.

[15] Qi X, Xing F, Foran DJ, Yang L (2012). Robust segmentation of overlapping cells in histopathology specimens using parallel seed detection and repulsive level set. IEEE Trans Biomed Eng (TBME).

[16] Veta M, Huisman A, Viergever MA, van Diest PJ, Pluim JP. Marker-controlled watershed segmentation of nuclei in h&e stained breast cancer biopsy images. In: Biomedical Imaging: From Nano to Macro, 2011 IEEE International Symposium On. IEEE: 2011. p. 618–21. 10.1109/isbi.2011.5872483.

[17] Loy G, Zelinsky A (2003). Fast radial symmetry for detecting points of interest. Pattern Anal Mach Intell IEEE Trans.

[18] Parvin B, Yang Q, Han J, Chang H, Rydberg B, Barcellos-Hoff MH (2007). Iterative voting for inference of structural saliency and characterization of subcellular events. IEEE Trans Image Process (TIP).

[19] Comaniciu D, Meer P (2002). Mean shift: a robust approach toward feature space analysis. IEEE Trans Pattern Anal Mach Intell (TPAMI).

[20] Krizhevsky Alex, Sutskever Ilya, Hinton Geoffrey E. (2017). ImageNet classification with deep convolutional neural networks. Communications of the ACM.

[21] Szegedy C, Liu W, Jia Y, Sermanet P, Reed S, Anguelov D, Erhan D, Vanhoucke V, Rabinovich A. Going deeper with convolutions. arXiv preprint. 2014. arXiv:1409.4842.

[22] Girshick R, Donahue J, Darrell T, Malik J. Rich feature hierarchies for accurate object detection and semantic segmentation. In: Computer Vision and Pattern Recognition (CVPR), 2014 IEEE Conference On. IEEE: 2014. p. 580–7. 10.1109/cvpr.2014.81.

[23] Erhan D, Szegedy C, Toshev A, Anguelov D. Scalable object detection using deep neural networks. In: Computer Vision and Pattern Recognition (CVPR), 2014 IEEE Conference On. IEEE: 2014. p. 2155–62. 10.1109/cvpr.2014.276.

[24] Farabet C, Couprie C, Najman L, LeCun Y (2013). Learning hierarchical features for scene labeling. Pattern Anal Mach Intell IEEE Trans.

[25] Cireşan Dan C., Giusti Alessandro, Gambardella Luca M., Schmidhuber Jürgen (2013). Mitosis Detection in Breast Cancer Histology Images with Deep Neural Networks. Medical Image Computing and Computer-Assisted Intervention – MICCAI 2013.

[26] Ciresan D, Giusti A, Schmidhuber J, et al.Deep neural networks segment neuronal membranes in electron microscopy images. In: NIPS: 2012. p. 2852–60.

[27] Song Youyi, Zhang Ling, Chen Siping, Ni Dong, Lei Baiying, Wang Tianfu (2015). Accurate Segmentation of Cervical Cytoplasm and Nuclei Based on Multiscale Convolutional Network and Graph Partitioning. IEEE Transactions on Biomedical Engineering.

[28] Xing F, Xie Y, Yang L (2016). An automatic learning-based framework for robust nucleus segmentation. IEEE Trans Med Imaging.

[29] Boykov Y, Kolmogorov V (2004). An experimental comparison of min-cut/max-flow algorithms for energy minimization in vision. Pattern Anal Mach Intell IEEE Trans.

[30] Long J, Shelhamer E, Darrell T. Fully convolutional networks for semantic segmentation. In: Proceedings of the IEEE Conference on Computer Vision and Pattern Recognition: 2015. p. 3431–40. 10.1109/cvpr.2015.7298965. 10.1109/TPAMI.2016.2572683 27244717

[31] Cox David R. (1992). Regression Models and Life-Tables. Springer Series in Statistics.

[32] Kaplan EL, Meier P (1958). Nonparametric estimation from incomplete observations. J Am Stat Assoc.

[33] Harrington DP, Fleming TR (1982). A class of rank test procedures for censored survival data. Biometrika.

[34] Otsu N (1975). A threshold selection method from gray-level histograms. Automatica.

[35] Carpenter AE, Jones TR, Lamprecht MR, Clarke C, Kang IH, Friman O, Guertin DA, Chang JH, Lindquist RA, Moffat J (2006). Cellprofiler: image analysis software for identifying and quantifying cell phenotypes. Genome Biol.

[36] Simon N, Friedman J, Hastie T, Tibshirani R (2011). Regularization paths for cox’s proportional hazards model via coordinate descent. J Stat Softw.

[37] Zou H, Hastie T (2005). Regularization and variable selection via the elastic net. J R Stat Soc Ser B (Stat Methodol).

[38] Sivic J, Zisserman A, et al.Video google: A text retrieval approach to object matching in videos. 10.1109/iccv.2003.1238663.

[39] Yang J, Yu K, Gong Y, Huang T. Linear spatial pyramid matching using sparse coding for image classification. In: Computer Vision and Pattern Recognition, 2009. CVPR 2009. IEEE Conference On. IEEE: 2009. p. 1794–801. 10.1109/cvpr.2009.5206757.

[40] Wang J, Yang J, Yu K, Lv F, Huang T, Gong Y. Locality-constrained linear coding for image classification. In: Computer Vision and Pattern Recognition (CVPR), 2010 IEEE Conference On. IEEE: 2010. p. 3360–7. 10.1109/cvpr.2010.5540018.

[41] Ronneberger Olaf, Fischer Philipp, Brox Thomas (2015). U-Net: Convolutional Networks for Biomedical Image Segmentation. Lecture Notes in Computer Science.

[42] Falk T, Mai D, Bensch R, Çiçek Ö, Abdulkadir A, Marrakchi Y, Böhm A, Deubner J, Jäckel Z, Seiwald K (2019). U-net: deep learning for cell counting, detection, and morphometry. Nat Methods.

[43] Gong Yunchao, Wang Liwei, Guo Ruiqi, Lazebnik Svetlana (2014). Multi-scale Orderless Pooling of Deep Convolutional Activation Features. Computer Vision – ECCV 2014.

[44] Oquab M, Bottou L, Laptev I, Sivic J. Learning and transferring mid-level image representations using convolutional neural networks. In: Proceedings of the IEEE Conference on Computer Vision and Pattern Recognition: 2014. p. 1717–24. 10.1109/cvpr.2014.222.

[45] Babenko A, Slesarev A, Chigorin A, Lempitsky V. Neural codes for image retrieval. In: European Conference on Computer Vision. Springer: 2014. p. 584–99.

[46] Zhou B, Khosla A, Lapedriza A, Oliva A, Torralba A. Learning deep features for discriminative localization. In: Proceedings of the IEEE Conference on Computer Vision and Pattern Recognition: 2016. p. 2921–9. 10.1109/cvpr.2016.319.

[47] Simonyan K, Zisserman A. Very deep convolutional networks for large-scale image recognition. arXiv preprint. 2014. arXiv:1409.1556.

[48] Lin M, Chen Q, Yan S. Network in network. arXiv preprint. 2013. arXiv:1312.4400.

[49] Dalal N, Triggs B. Histograms of oriented gradients for human detection. In: Computer Vision and Pattern Recognition, 2005. CVPR 2005. IEEE Computer Society Conference On, vol. 1. IEEE: 2005. p. 886–93. 10.1109/cvpr.2005.177.

[50] Csurka G, Dance C, Fan L, Willamowski J, Bray C (2004). Visual categorization with bags of keypoints[C]. Workshop on statistical learning in computer vision. ECCV.

[51] Tibshirani Robert (2011). Regression shrinkage and selection via the lasso: a retrospective. Journal of the Royal Statistical Society: Series B (Statistical Methodology).

[52] Harrell FE, Califf RM, Pryor DB, Lee KL, Rosati RA (1982). Evaluating the yield of medical tests. Jama.

[53] Jia Y, Shelhamer E, Donahue J, Karayev S, Long J, Girshick R, Guadarrama S, Darrell T. Caffe: Convolutional architecture for fast feature embedding. In: Proceedings of the 22nd ACM International Conference on Multimedia. ACM: 2014. p. 675–8. 10.1145/2647868.2654889.

